# Efficacy of low-dose intra-articular tranexamic acid in total knee replacement; a prospective triple-blinded randomized controlled trial

**DOI:** 10.1186/1471-2474-14-340

**Published:** 2013-12-05

**Authors:** Paphon Sa-ngasoongsong, Siwadol Wongsak, Pongsthorn Chanplakorn, Patarawan Woratanarat, Supaporn Wechmongkolgorn, Bussanee Wibulpolprasert, Pornchai Mulpruek, Viroj Kawinwonggowit

**Affiliations:** 1 Department of Orthopedics, Faculty of Medicine, Ramathibodi Hospital, Mahidol University, 270, Rama VI Road, Ratchathewi, Bangkok, Thailand10400; 2 Department of Nursing, Faculty of Medicine, Ramathibodi Hospital, Mahidol University, Bangkok, Thailand; 3 Department of Radiology, Faculty of Medicine, Ramathibodi Hospital, Mahidol University, Bangkok, Thailand

**Keywords:** Intra-articular tranexamic acid, Blood loss reduction, Blood transfusion reduction, Clamp drain technique

## Abstract

**Background:**

Recently, a number of studies using intra-articular application of tranexamic acid (IA-TXA), with different dosage and techniques, successfully reduced postoperative blood loss in total knee replacement (TKR). However, best of our knowledge, the very low dose of IA-TXA with drain clamping technique in conventional TKR has not been yet studied. This study aimed to evaluate the effectiveness and dose-response effect of two low-dose IA-TXA regimens in conventional TKR on blood loss and blood transfusion reduction.

**Methods:**

Between 2010 and 2011, a triple-blinded randomized controlled study was conducted in 135 patients undergoing conventional TKR. The patients were allocated into three groups according to intra-articular solution received: Control group (physiologic saline), TXA-250 group (TXA 250 mg), and TXA-500 group (TXA 500 mg). The solution was injected after wound closure followed by drain clamping for 2 hours. Blood loss and transfusion were recorded. Duplex ultrasound was performed. Functional outcome and complication were followed for one year.

**Results:**

There were forty-five patients per groups. The mean total hemoglobin loss was 2.9 g/dL in control group compared with 2.2 g/dL in both TXA groups (*p* > 0.001). Ten patients (22%, control), six patients (13%, TXA-250) and none (TXA-500) required transfusion (*p* = 0.005). Thromboembolic events were detected in 7 patients (4 controls, 1 TXA-250, and 2 TXA-500). Functional outcome was non-significant difference between groups.

**Conclusions:**

Combined low-dose IA-TXA, as 500 mg, with 2-hour clamp drain is effective for reducing postoperative blood loss and transfusion in conventional TKR without significant difference in postoperative knee function or complication.

**Trial registration:**

ClinicalTrials.gov NCT01850394.

## Background

Extensive perioperative blood loss (PBL) requiring blood transfusion is one of the most important factors concerning by many surgical specialists due to risk of bleeding-related complication and transfusion-related morbidity [1,2]. Among the orthopedic interventions, total knee replacement (TKR) is a major surgery associated with considerable amount of blood loss, and therefore, resulting in a significant number of patients needed transfusion requirement. Regarding of the proven methods used for reducing PBL in TKR, tranexamic acid (TXA), an anti-fibrinolytic agent, has been demonstrated ability to decrease PBL and proportion of patients required postoperative blood transfusion and could be applied via either intravenous application [3] or intra-articular injection [4]. However, the intravenous application of TXA has a major disadvantage on uncertain risk of drug-induced venous thromboembolic (VTE) complication from prolonged high systemic drug level especially with multiple injection or continuous infusion regimens [5]. Thus, for safety concern of the VTE complication, there has been a growing attention of using TXA as intra-articular agent (IA-TXA) in TKR [4,6-12].

Several studies demonstrated an efficacy of the IA-TXA application to reduce postoperative blood loss and knee swelling in conventional TKR (Con-TKR); however, there still has been no guideline for using of IA-TXA such as the dosage of TXA which varied from 500 mg to 3,000 mg, using of drain, time interval of drain clamping, and the use of concomitant carbazochrome sodium sulfate mixed into the intra-articular solution [6-8,10-13]. Moreover, the absorption of TXA into blood had been shown to be above therapeutic concentration [12]. Recently Sa-ngasoongsong et al., developed a new IA-TXA method, combined low dose of TXA (as 250 mg) with 2-hour clamp drain, for reducing blood loss and transfusion in computer-assisted surgery total knee replacement (CAS-TKR) without any significant complication [9]. This technique should also be able to use in conventional TKR that carries risk of higher blood loss.

The objective of this study included the following: (1) whether low dosage IA-TXA solution was able to reduce PBL and need of blood transfusion in conventional TKR and if it can then, (2) how the efficacy of IA-TXA does depend on the dosage of TXA used?

## Methods

This was a single-centered, prospective triple-blinded randomized study with 1:1:1 allocation ratio and was approved by Committee on Human Right Related to Researches Involving Human Subjects (Protocol number ID 01-53-06). Informed consent was obtained from all patients who participated in this study, before the surgery was scheduled, in accordance with the Declaration of Helsinki. The manuscript was prepared according to the Consolidated Standards of Reporting Trials (CONSORT) 2010 guideline [14].

The inclusion criterion was the patients diagnosed as primary knee osteoarthritis and underwent unilateral primary cemented Con-TKR between January 2010 and January 2011. The exclusion criteria were (1) no risk of abnormal bleeding tendency or bleeding disorder (normal coagulogram, serum creatinine < 2.0 mg/dL, stop nonsteroidal anti-inflammatory drugs and antiplatelet drugs more than 7 days; and (2) no contra-indication for TXA use (no active intravascular clotting process, no acquired defective colour vision, no subarachnoid hemorrhage, no hypersensitivity to TXA, and no any of history of serious adverse effects, thrombotic disorder and hematuria).

**Figure 1:**
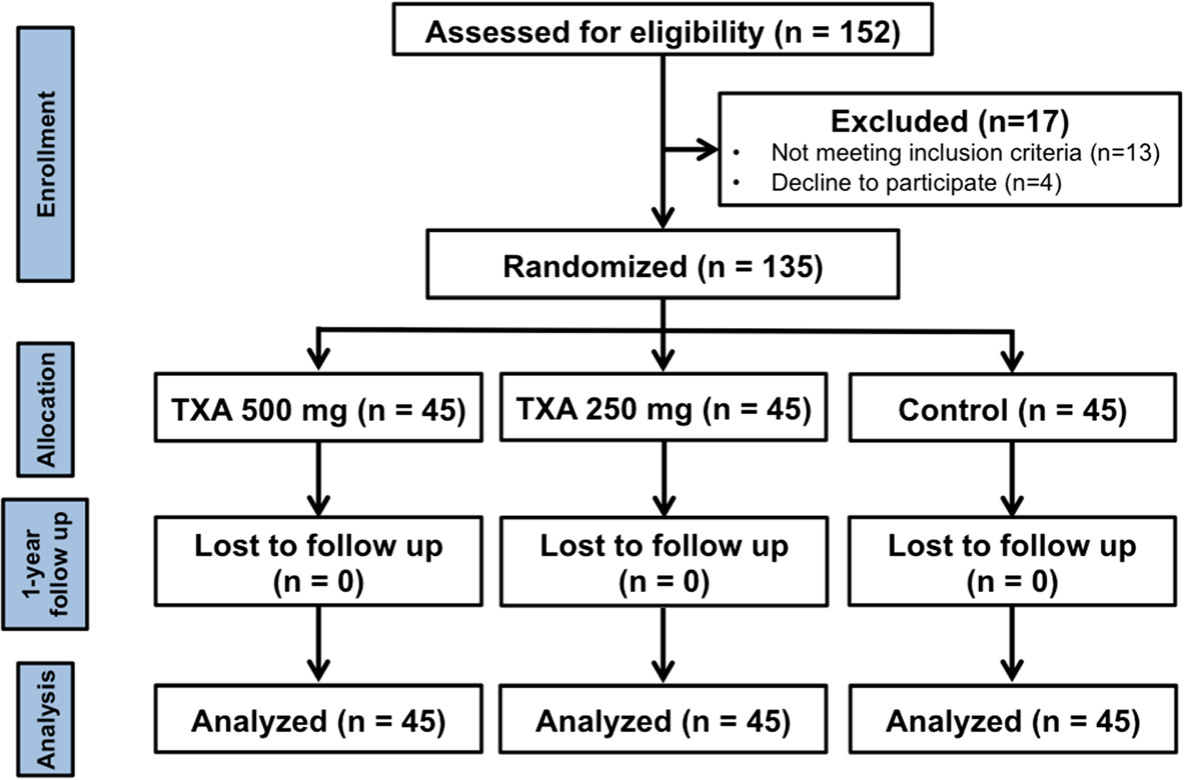
Illustration showed the flow diagram of this study.

The blocked-randomization was generated by STATA 11.0 software (Stata Corp, College Station, Texas, USA), with block size of nine, and further concealed with sealed envelopes in the sequentially numbered container. After fascia closure, one of our authors (Su.W.), who did not involve in the operation and in outcome assessment, was responsible for opening these envelopes and preparing the study medication. Then all patients were allocated to one of three groups; control group, TXA-250 group or TXA-500 group (Figure 1).

All of the solution in this study was prepared as total 25-ml volume in the same-size syringe, according to allocation received, under sterile condition and behind the fluoroscopic scene in order to blind surgeon and outcome assessor. Due to a fixed concentration of TXA medication (250 mg per 5 ml, Transmin®, OLIC (Thailand) Limited, Ayutthaya, Thailand), the dosage of TXA could be calculated from the volume of TXA used. In TXA-250 and TXA-500 group, the solution contained 250-mg (5 ml) or 500-mg (10 ml) of TXA respectively, and then were added with physiologic saline to create a total volume of 25 ml. In the control group, the solution was 25 ml of physiologic saline. All of prepared solution had the same appearance. This solution was injected to the knee joint via drain tube after fascia closure, in order to prevent leakage. Then the vacuum drain was connected to the drain tube and clamped for 2 hours.

Demographic data such as age, gender, height, weight, underlying disease, side of operation, American Society of Anesthesiologists (ASA) physical status [15], preoperative hemoglobin (Hb) and hematocrit (Hct) were collected preoperatively, by research assistant, and then further calculated into body mass index (BMI) and estimated blood volume (EBV) [16]. Preoperative mechanical axis and baseline knee function score, as Knee Society Knee score (KSK) and Western Ontario and McMaster Universities Osteoarthritis Index (WOMAC) were obtained [17,18].

The surgery was performed by one of the authors (V.K.), who was an experienced arthroplasty surgeon. The decision on anesthetic technique, general or regional anesthesia, depended on the anesthesiologists who did not involve in the study. The prostheses used in this present study were Nexgen® total knee system (Zimmer Inc, Warsaw, Indiana, USA). Due to awareness of sex-related difference in antero-posterior diameter of the distal femur, Nexgen® Gender prosthesis was specifically used in female patient while Nexgen® Flex prosthesis was used in male patient. The pneumatic tourniquet was applied at proximal thigh with pressure as 350 mmHg. The surgical approach was midline skin incision, medial parapatellar arthrotomy and midvastus incision. After the bony structure was prepared, all prosthesis components were inserted with full cementation (Palacos®, Hevaeus Medical GmbH, Germany). A standard drain tube (size 8 Redon drain, B-Braun Ltd.) was placed deep into knee joint, exited superolaterally, and connected to high pressure vacuum drain (Drainobag® 600V Lock, B-Braun, Melsungen AG, Germany). No superficial drain was used in this study. After fascia closure, one of our authors (Su.W.), was assigned to open the sealed envelopes and prepare the solution in 25-ml syringe behind the scene under sterile technique in order to blind the surgeon. Then the prepared sterile solution, either placebo or TXA solution, was sent to the surgeon and further injected into knee joint. Subcutaneous and skin closure was performed subsequently. Bulky compressive dressing was applied and the drain was clamped before tourniquet was deflated. The 2-hour period of drain clamping and the time of drain opening were then written on the drain bottle and further informed to the well-trained research assistant, who was blinded to group allocation. At the ward, the research assistant was responsible for fully opening the drain at the setting time and recording the amount of drain volume as data collection protocol.

**Figure 2:**
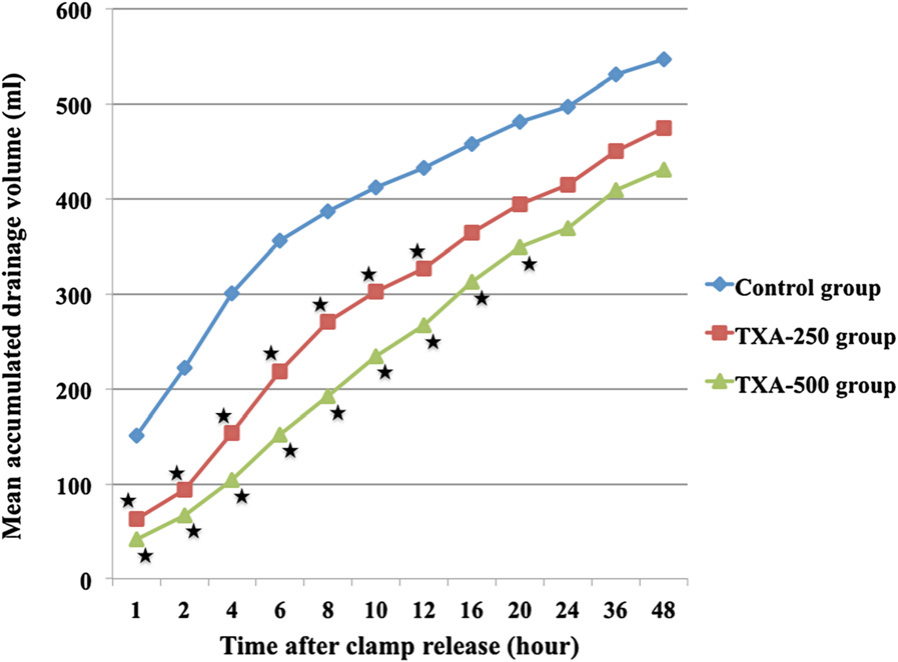
**The mean accumulated drainage volume (ADV) at each time point throughout 48-hour drainage recorded period, in all three groups, demonstrated a significant reduction in both TXA groups only the early period after clamp release (the first 12-hour period in TXA-250 group and the first 20-hour period in TXA-500 group) (**★ **=** ***p*** **< 0.05).**

Basic postoperative data, such as drainage volume, Hct, Hb, amount of blood transfusion, and WOMAC score, were collected by a well-trained research assistant who was blinded to the group allocation and did not involve in the medication preparation process. Postoperative data requiring clinical examination or physician diagnosis, such as range of motion, and diagnosis of complication, were collected by one of the authors. Both of them were blinded to the treatment allocation. Accumulated drain volume (ADV) was recorded by research assistant at 1, 2, 4, 6, 8, 10, 12, 16, 20, 24, 36, and 48 hours after clamp release (Figure 2). Drain re-clamp protocol was used to prevent severe immediate brisk blood loss by re-clamping the drain, for 30 minutes, if drainage blood loss after release was more than 250 ml/hour in the first hour and then recorded as incidence of re-clamping. If the wound had excessive bleeding detected by bloody oozing on compressive dressing, the dressing was changed and recorded as incidence of re-dressing. These re-clamping and re-dressing events were managed and recorded by the research assistant who did not know the allocation result. Postoperative Hct was measured immediately at ward and subsequently at the fourth hour, then every 8 hours on the first day, every 12 hours on the second day and once daily until the fourth postoperative day. If Hct level was less than 25%, Hb was measured in order to evaluate the need of transfusion. According to ASA guideline, blood transfusion was considered when Hb was less than 8 gm% or the patient had positive anemic symptom (dyspnea, tachypnea and hypoxemia) [19]. On the fourth postoperative day, Hb was measured in order to calculate for total blood loss.

Postoperative blood loss was measured in three different methods same as our previous study [9]: drainage blood loss (DBL), total Hb loss (THL) and calculated total blood loss (CTBL). THL and CTBL were calculated by using specific formulation [16,20]. Drainage volume increment per hour was further calculated, as drainage blood loss rate (DBLR), by dividing DBL increment in each time interval with number of hours in that time interval [9].

Blood transfusion requirement was measured by recording the number of patients receiving transfusion and amount of blood transfusion in unit.

The patients were all sent to document deep vein thrombosis (DVT) by duplex ultrasound, performed by a senior radiologist (B.W.), on the fourth postoperative day. If the patients developed postoperative clinical presentation suspected pulmonary embolism (PE) such as acute dyspnea or unexplained hypoxemia, the computer tomographic angiogram was then performed to confirm the diagnosis by the same senior radiologist. The incidence of DVT and PE was recorded as VTE complication.

Functional outcomes, such as KSK and WOMAC score, were evaluated at the clinic at 3-month, 6-month and 1-year period postoperatively.

Postoperative complications such as wound hematoma, surgical site infection or systemic infection were evaluated at ward, at clinic as time of follow-up and/or by phone interview periodically.

Statistical analysis was performed using Stata software version 11.0 (Stata Corp, College Station, Texas, USA). Intention-to-treat analysis was applied. Normality of data was tested by Kolmogorov-Smirnov test. Continuous data were presented as mean and standard deviation, and compared with one-way analysis of variance (ANOVA). The comparison of drainage blood loss parameters between groups according to time was performed by repeated measurement with Scheffe test post hoc analysis. Categorical data were presented as proportion and compared with Chi-square test. Significant difference was considered if *p* value < 0.05.

Sample size estimation was calculated by using data review on hemoglobin loss in our patients who underwent primary Con-TKR before this study (mean hemoglobin loss ± standard deviation = 3.3 ± 1.0 g/dl). We assumed that this IA-TXA application should reduce blood loss more than 20% compared with control group. Setting the pre-study power of test (β) as 0.8, significant difference (α) as 0.05, and the ratio of sample size in each group as 1:1:1; therefore, the sample size of each group was 45 patients.

## Results

From January 2009 to January 2010, there were 152 patients that underwent primary unilateral Con-TKR participated into this study. Seventeen patients were excluded that including refusal to participate (4 patients), serum creatinine more than 2.0 mg% (4 patients), rheumatoid arthritis (4 patients), abnormal coagulogram (3 patients), and history of stroke (2 patients). A total of 135 patients was recruited and then equally randomized into each group (45 patients per group). All patients had completed 1-year follow-up examination (Figure 1). Demographic data were shown in Table 1. There was no significant difference between all three groups in terms of age, sex, BMI, EBV, number of diabetes patients, ASA physical status, side of operation, preoperative mechanical axis, preoperative functional score, anesthetic technique, operative time, and incision size. However, TXA-500 group showed significantly higher in preoperative Hb level compared with TXA-250 group (*p* = 0.02).

**Table 1:** The patients’ preoperative characteristics data

	**Control group**	**TXA-250 group**	**TXA-500 group**	***p*-value**
	**(n = 45)**	**(n = 45)**	**(n = 45)**	
**Demographic characteristics**				
Age✝ (year)	66.2 (7.3)	67.6 (8.7)	68.1 (6.2)	0.44
Female gender◆	43 (95.6%)	42 (93.3%)	40 (88.9%)	0.47
Body mass index✝ (kg/m2)	26.3 (3.7)	26.2 (3.7)	27.3 (4.7)	0.37
Estimated blood volume✝ (ml)	3146 (310)	3090 (246)	3227 (401)	0.14
DM◆	34 (75.6%)	36 (80.0%)	34 (75.6%)	0.9
ASA classification (II/III) (no. of patients)	29/16	20/25	20/25	0.1
**Preoperative laboratory values**				
Preoperative hemoglobin✝ (g/dL)	12.1 (1.1)	11.9 (1.0)	12.6 (1.3)*^c^	0.02*
**Surgical characteristics**				
Regional anesthesia◆	42 (93%)	39 (87%)	43 (96%)	0.38
Right side◆	26 (57.8)	25 (55.6)	23 (51.1)	0.86
Preoperative mechanical axis◼	4.9 varus (15 varus to 8 valgus)	5.0 varus (16 varus to 13 valgus)	5.2 varus (20 varus to 6 valgus)	0.97
Operative time✝ (minute)	79.1 (14.3)	78.8 (13.0)	81.1 (13.1)	0.65
Surgical incision✝ (cm)	12.5 (2.2)	12.3 (2.2)	13.2 (1.2 )	0.09
**Functional characteristics**				
Preoperative KSK Score✝	77.3 (11.0)	76.8 (11.9)	76.4 (13.3)	0.94
Preoperative WOMAC score✝	59.8 (9.5)	60.3 (9.8)	60.0 (9.7)	0.98

✝ = The values were presented as mean (standard deviation), ◼ = The values were presented as mean (range).

◆ = The values were presented as number of patients having that condition (percentage of this group).

*TXA*; tranexamic acid, DM; patient having underlying diabetes mellitus, ASA; American Society of Anesthesiologists.

*KSK*; Knee Society Knee, *WOMAC*; Western Ontario and McMaster Universities Osteoarthritis Index.

* = p <0.05; ^c^ = comparison between TXA-250 and TXA-500 group with p < 0.05.

The mean THL and CTBL in TXA-500 and TXA-250 groups were significantly lower compared to control group (*p* <0.001 both). However, the mean DBL in both TXA groups were non-significantly lower than control group (*p* = 0.09). Allogeneic blood transfusion in TXA-500 group (none) was also significantly lower than TXA-250 group (six patients) and control group (ten patients) (*p* = 0.005) (Table 2).

**Table 2:** Blood loos and blood transfusion outcome in all three groups

	**Control group**	**TXA 250 mg**	**TXA 500 mg**	***p*-value**
	**(n = 45)**	**(n = 45)**	**(n = 45)**	
**Blood loss outcome variables**				
Drainage blood loss✝ (ml)	546.9 (273.0)	475.0 (254.4)	430.2 (224.0)	0.09
Total hemoglobin loss✝ (g/dl)	2.9 (1.2)	2.2 (0.7)**^a^	2.2 (0.7)**^b^	< 0.001**
Calculated total blood loss✝ (ml)	329.2 (119.4)	239.7 (83.7)**^a^	217.2 (86.1)**^b^	< 0.001**
**Packed red blood-cell transfusion**				
Blood transfusion use◆	10 (22%)	6 (13%)	0 (0%)**	0.005*

✝ = The values were presented as mean (standard deviation); *, p < 0.05; **, p < 0.001.

◆ = The values were presented as number of patients having that condition (percentage of this group).

a = comparison between control and TXA-250 group with p < 0.05.

b = comparison between control and TXA-500 group with p < 0.05.

**Figure 3:**
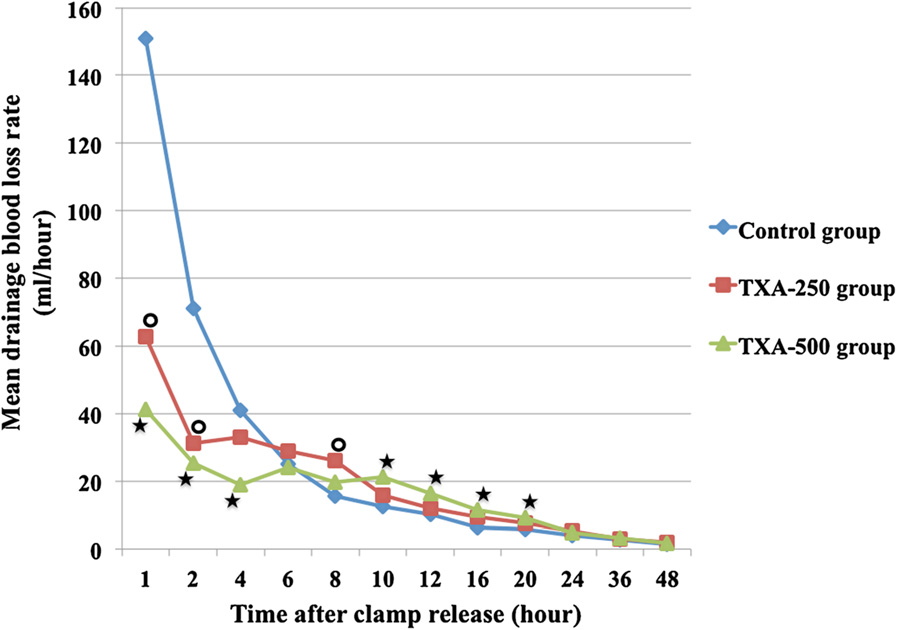
**The drainage blood loss rate (DBLR) at each time point throughout 48-hour drainage recorded period, in all three groups, showed a significant reduction in both TXA groups compared to control group in the early period after clamp release (the first 2-hour period in TXA-250 group and the first 4-hour period in TXA-500 group (**★ **=** ***p*** **< 0.05).** After that, the mean DBLR in both TXA groups were decreased at a slower rate than those in control group resulting in unexpected significant greater in the mean DBLR in both TXA groups compared to control group for several hours (★ = *p* < 0.05).

The effects of two TXA regimens on blood loss reduction were demonstrated in Figures 2 and 3. TXA-500 group had the greatest reduction in the mean ADV at every time point and these values were significantly lower than control group (*p* <0.05), from the first hour to the twentieth hour after clamp release. TXA-250 group showed significantly lower in the mean ADV (*p* <0.05) than control group only from the first hour to the twelfth hour after clamp release (Figure 2). TXA-500 group also had the greatest and significant reduction (*p* <0.05) in the mean DBLR compared to control group during the first 4-hour period after clamp release, while those in TXA-250 group were significantly lower (*p* <0.05) than control group only for the first 2-hour period after clamp release (Figure 3).

WOMAC and KSK scores were significantly improved postoperatively in all three groups throughout 1-year follow-up period (p <0.001 both), and no significant difference between groups was detected in both scores (*p* = 0.42 and 0.91, respectively). The incidence of re-clamping and re-dressing in TXA-500 group were significantly lower than other two groups (*p* =0.01, 0.05 respectively). There was no significant difference in VTE complications (1 symptomatic PE and 3 DVT in control group, 1 DVT in TXA-250 group, and 2 DVT in TXA-500 group, *p* = 0.35). None of postoperative infection was detected in this study (Table 3).

**Table 3:** Functional outcome and postoperative complications in all three groups

	**Control group**	**TXA 250 mg**	**TXA 500 mg**	***p*-value**
	**(n = 45)**	**(n = 45)**	**(n = 45)**	
**WOMAC score**✝				
3 months	36.7 (8.2)**	35.8 (7.6)**	35.5 (7.2)**	0.42
6 months	25.4 (6.0)**	23.1 (6.5)**	23.5 (6.6)**	
1 year	15.5 (6.6)**	15.1 (6.2)**	14.5 (7.1)**	
*p*-value within group	< 0.001**	< 0.001**	< 0.001**	
**Knee society knee score**✝				
3 months	121.7 (10.2)**	121.1 (10.1)**	123.3 (9.1)**	0.91
6 months	141.0 (11.5)**	141.2 (9.4)**	140.0 (8.9)**	
1 year	148.9 (10.1)**	151.2 (9.1)**	150.9 (9.3)**	
*p*-value within group	< 0.001**	< 0.001**	< 0.001**	
**Complications**◆				
Re-clamp	6 (13%)	1 (2%)	0 (0%)*^b^	0.01
Re-dressing	3 (7%)	0 (0%)*^a^	0 (0%)*^b^	0.05
VTE	4 (9%)	1 (2%)	2 (4%)	0.35
Congestive heart failure	0 (0%)	1 (2%)	0 (0%)	1.00

✝ = The values were presented as mean (standard deviation), ◆ = The values were presented as number of patients having that condition (percentage of this group), **; p-value between time < 0.001, *; p < 0.05.

a = comparison between control and TXA-250 group with p < 0.05.

b = comparison between control and TXA-500 group with p < 0.05.

Furthermore, there was an unexpected finding in this study. While the mean DBLR in control group, after the first 4-hour period, was descended in an exponentially fashion, those in both TXA groups were decreased at a slower rate. Consequently, a significant increase of the mean DBLR in both TXA groups, compared to control group, was demonstrated in a period between 8 and 20 hours (p < 0.05) (Figure 3).

## Discussion

Recently, intra-articular tranexamic acid (IA-TXA) application has become one of most popular methods for reducing blood loss and transfusion requirement in conventional TKR (Con-TKR) [6-12]. However, to our knowledge, previous studies demonstrated ability to reduce postoperative blood loss with a variety of techniques and mostly using high dosage of tranexamic acid (TXA) (500-3,000 mg) [6,7,10-12], while our previous study also showed benefit of blood loss and transfusion reduction in computer-assisted surgery TKR with combined low-dose TXA (250 mg) with 2-hour clamp drain [9]. In this prospective randomized controlled study, we aimed to evaluate the efficacy of two regimens of low-dose IA-TXA (250 mg and 500 mg) combined with 2-hour clamp drain, and the effect of the different dosage on the reduction of blood loss and transfusion.

This present study had some limitations. First, although our result showed an ability to decrease blood loss and transfusion, it still needed more study population to identify any rare possible complications such as symptomatic DVT or PE. Second, our study did not measure serum TXA; however, the expected systemic absorption should be low due to the low dosage of TXA and the use of high negative pressure drainage system as demonstrated in our result that the incidence of VTE was no statistical difference compared to control.

Our result demonstrated that intra-articular application with low-dosage TXA, as 250 and 500 mg, was effective for reducing postoperative blood loss in which comparable with the result from previous studies that used TXA as a single topical blood conservative agent (Table 4). Although the DBL in both TXA groups significantly reduced only in early period after clamp release compared with control group (Figure 2), this result also comparable with the previous studies in Con-TKR [6,7]. However, two studies [10,11] reported the significant reduction in the total DBL between control group and IA-TXA group. This discrepancy between our data and those studies might be explained by three reasons. First, our sample size in this study was unable to detect a less-than 20% difference in DBL between both TXA groups and control group. Secondly, the exact DBL in control group should be higher than the presented value in Table 3 due to drain re-clamping in 6 patients (13%) following our blood conservative protocol. Finally, some surgical techniques in previous studies might enhance the efficacy of IA-TXA method such as using high TXA dosage in minimally invasive surgery TKR [11] and combined intraoperative hemostasis with tourniquet release before wound closure [10]. Moreover, our data also demonstrated the ability to reduce blood transfusion requirement as comparable with Seo et al. [11] and Roy et al. [10] studies.

**Table 4:** Comparison of the previous RCT studies using tranexamic acid (TXA) as a single intra-articular agent with this study

**Studies**	**TXA dosage (g)**	**Bone cutting guide**	**Prosthesis**	**Technique used**	**DBL**		**THL**	**CTBL**	**BT**
					**Early**❖	**Total**			
Wong (2010) [12]	1.5 & 3.0	IM	Triathlon, Genesis II	5 min T.A., no drain	n/a	n/a	Sig	Sig	NS
Maniar (2012) [7]	3.0	EM	PFC Σ	5 min T.A., clamp drain♯ 2 hr then fully open	Sig	NS	n/a	Sig	NS
Ishida (2011) [6]	2.0	IM	Advance, Nexgen, Scorpio, Vanguard, PFC Σ	IA, clamp drain 30 min then gradually open	Sig	NS	Sig	Sig	NS
Seo (2012) [11]	1.5	EM	Scorpio	IA, with non-clamp drain	n/a	Sig	Sig	n/a	Sig
Roy (2012) [10]	0.5	IM	Scorpio	IA, clamp drain 1 hr then fully open	Sig	Sig	Sig	n/a	Sig
Sa-ngasoongsong (2011) [9]	0.25	EM	LCS, PFC Σ	IA, clamp drain 2 hr then fully open	Sig	Sig	Sig	Sig	Sig
This study	0.25, 0.5	IM	Nexgen	IA, clamp drain 2 hr then fully open	Sig.	N-S	Sig.	Sig.	Sig

*TXA*; Tranexamic Acid, *DBL*; drainage blood loss, ❖; accumulated DBL in early period (6-24 hours after surgery).

*THL*; total hemoglobin loss, *CTBL*; calculated total blood loss, *BT*; blood transfusion.

IM; intramedullary femoral cutting guide, EM; extramedullary femoral cutting guide.

*T.A*; Topical application, *IA*; intra-articlar, min; minute, hr; hour, ♯; intra-articular drain.

n/a; not available, *Sig*; significant reduction compared with control group, NS; non-significant difference compared with control group.

Regards to the effect of different dosage, our result also demonstrated the greater ability to reduce blood loss and blood transfusion in TXA-500 group compared with TXA-250 group (Figures 2 and 3). The 500-mg dosage significantly reduced in both ADV and DBLR with higher degree and also had a longer effect on DBLR compared to the 250-mg dosage in the early period after drain opening. This dose-dependent effect also demonstrated in Wong et al. study [12] that used 5-minute topical application of 1.5-g and 3.0-g TXA before wound closure without using drain. Although DBL was unavailable to compare, the result on THL and CTBL, between our study and Wong et al. study, were comparable (as 27% and 34% reduction in mean CTBL in TXA-250 and TXA-500 group respectively in this present study compared with 20% and 25% reduction in mean CTBL in 1.5-g and 3.0-g group in Wong et al. study). However, no significant difference in blood transfusion requirement between groups was found in Wong et al. [12] and Maniar et al. [7] studies, which used 5-minute topical application of 3.0-g TXA. The effect on blood transfusion reduction between the low-dose IA-TXA application in this present study and the high-dose TXA topical application in previous studies [7,12] might be explained by the “tissue contact time” or the time that TXA solution applied on the surgical bed. Compared to those previous topical TXA application studies, our study had a longer tissue contact time (2 hours in our study versus 5 minutes in Wong et al. and Maniar et al. study [7,12]). The prolonged tissue contact time in our study, due to using clamping drain technique, could enhance the effect of low-dose IA-TXA resulting in more effective in controlling postoperative blood loss in early period, and better efficacy on blood transfusion reduction.

Our study also demonstrated an unexpected finding of unique pattern of DBLR in both TXA groups that had decreased with a slower rate and resulted in a significant increase in DBLR between 8 and 20 hours compared to control group (Figure 3). This finding might be explained by two possible reasons. First topical application of IA-TXA with 2-hour drain clamping would result in a significant greater thrombus formation than control group that might produce more drainage volume from clot lysis. Second, an abrupt reduction in the TXA amount in the surgical wound (from clamp release) might result in a relative sudden activation of local fibrinolytic enzyme, such as plasminogen and plasmin. Consequently, the suddenly activated fibrinolysis would lead to a rapid clot lysis, or even re-bleeding from small vessels in the surgical wound.

## Conclusion

In conclusion, the combined intra-articular application of low-dose tranexamic acid, as 500 mg, with 2-hour clamping drain technique is effective for reducing postoperative blood loss and transfusion requirement in conventional total knee replacement without significant increase in postoperative complication.

## Abbreviations

PBL: Perioperative blood loss; TXA: Tranexamic acid; IA-TXA: Intra-articular tranexamic acid; TKR: Total knee replacement; Con-TKR: Conventional total knee replacement; CAS-TKR: Computer-assisted surgery total knee replacement; ASA: American society of anesthesiologists; Hb: Hemoglobin; Hct: Hematocrit; BMI: Body mass index; EBV: Estimated blood volume; KSK: Knee society knee; WOMAC: Western Ontario and McMaster universities osteoarthritis index; ADV: Accumulated drainage volume; DBL: Drainage blood loss; THL: Total hemoglobin loss; CTBL: Calculated total blood loss; DBLR: Drainage blood loss rate; DVT: Deep vein thrombosis; PE: Pulmonary embolism; VTE: Venous thromboembolic.

## Competing interests

All of the authors declare that they have no conflict of interest.

## Authors’ contributions

PS, M.D.^1^: Main researcher who designed and performed study, and prepare the manuscript; SW, M.D.^1^: Arthoplasty surgeon who assisted in research process; PC, M.D.^1^: Orthopaedic surgeon who helped in manuscript preparation; PW, M.D., Ph.D.^1^: Orthopaedic surgeon and experts in epidemiology who advised the study design and methods; SW^2^: Orthopaedic nurse who opened the randomized seal-envelopes and prepare the intra-articular solution; BW, M.D.^3^: Radiologist who performed all of the duplex doppler ultrasound; PM, M.D., MCh Orth^1^: Senior arthroplasty surgeon who assisted in research process; Viroj Kawinwonggowit, M.D.^1^: Senior arthroplasty surgeon and corresponding author who performed all of the operations. All authors read and approved the final manuscript.

## Authors’ information

P.S. and P.C. are orthopaedic surgeons who experienced and interested in intra-articular tranexamic acid injection methods with previous publication of this technique and work in Department of Orthopedics, Faculty of Medicine, Ramathibodi Hospital, Mahidol University.

V.K., P.M., and S.W. are experienced arthroplasty surgeon in Department of Orthopedics, Faculty of Medicine, Ramathibodi Hospital, Mahidol University.

P.W. is an orthopaedic surgeon and expert in epidemiology who work in Department of Orthopedics, Faculty of Medicine, Ramathibodi Hospital, Mahidol University.

Su.W. is an assistant operative nurse who interested in intra-articular tranexamic acid injection methods with previous publication of this technique and works in Orthopedic operative section, Ramathibodi Hospital.

B.W. is an expert in duplex ultrasonography in Department of Radiology, Faculty of Medicine, Ramathibodi Hospital, Mahidol University.

## Pre-publication history

The pre-publication history for this paper can be accessed here:

http://www.biomedcentral.com/1471-2474/14/340/prepub
